# Supplementary material for: DMRforPairs: identifying Differentially Methylated Regions between unique samples using array based methylation profiles
Source: BMC Bioinformatics. 2014 May 15;15:141. doi: 10.1186/1471-2105-15-141 (PMC4046028; doi:10.1186/1471-2105-15-141)
Supplement: Additional file 2 — DMRforPairs output for the comparison of A431-MCF7 and NA17018-NA17105. Please start from the HTML files in each folder. Available via the BMC Bioinformatics website. [file 1471-2105-15-141-S2.zip › 1394847754114233_MOESM2_ESM/A431_MCF7/figures/40283.pdf]

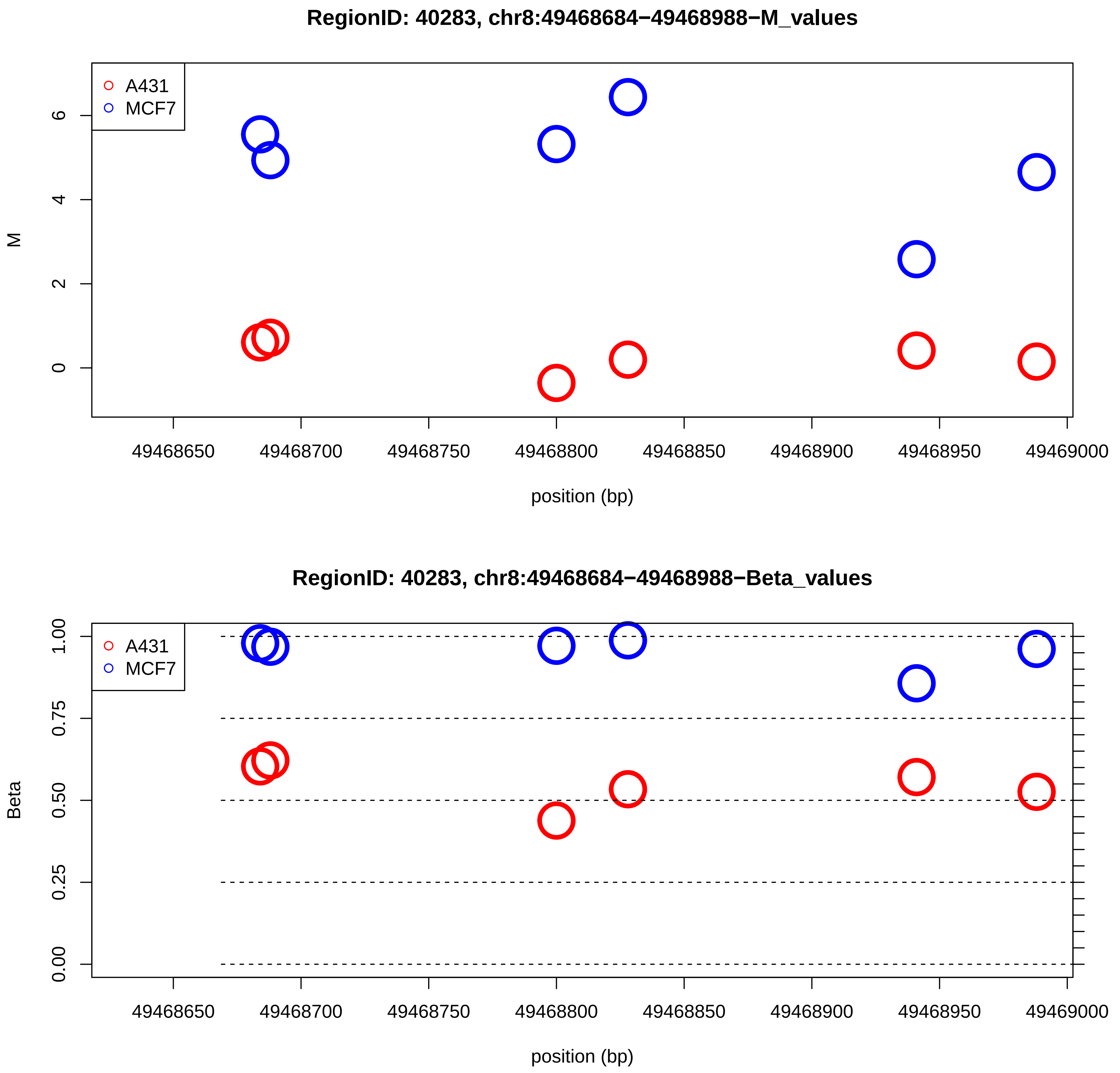
RegionID: 40283, chr8:49468684−49468988−M_values
A431
MCF7
M
6
4
2
0
49468650
49468700
49468750
49468800
49468850
49468900
49468950
49469000
position (bp)
RegionID: 40283, chr8:49468684−49468988−Beta_values
A431
MCF7
Beta
1.00
0.75
0.50
0.25
0.00
49468650
49468700
49468750
49468800
49468850
49468900
49468950
49469000
position (bp)

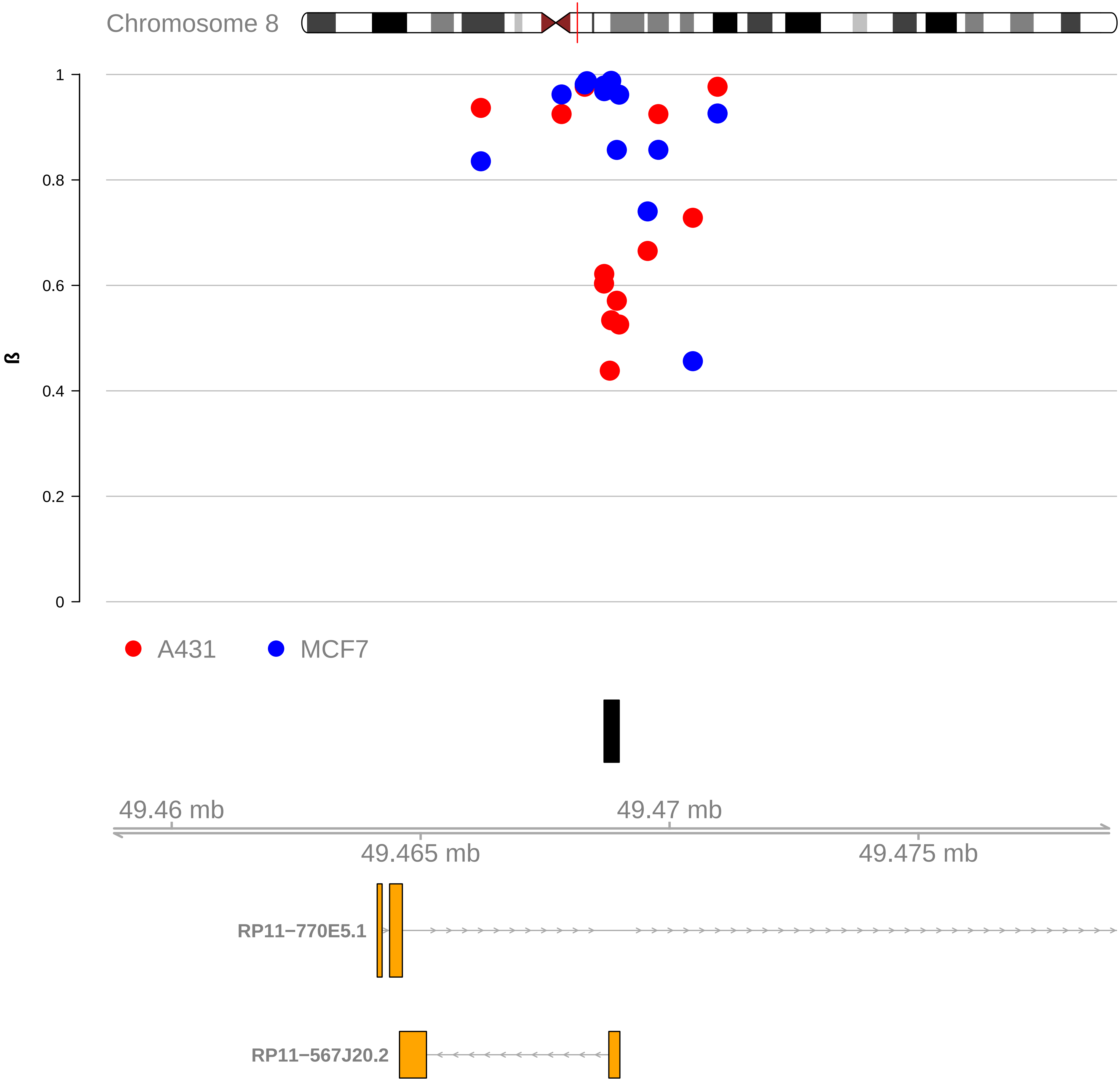
Chromosome 8
ß
1
0.8
0.6
0.4
0.2
0
A431
MCF7
49.46 mb
49.47 mb
49.465 mb
49.475 mb
RP11−770E5.1
RP11−567J20.2
